# Neither Tumor-Infiltrating Lymphocytes nor Cytotoxic T Cells Predict Enhanced Benefit from Chemotherapy in the DBCG77B Phase III Clinical Trial

**DOI:** 10.3390/cancers14153808

**Published:** 2022-08-05

**Authors:** Elahe Shenasa, Elisabeth Specht Stovgaard, Maj-Britt Jensen, Karama Asleh, Nazia Riaz, Dongxia Gao, Samuel Leung, Bent Ejlertsen, Anne-Vibeke Laenkholm, Torsten O. Nielsen

**Affiliations:** 1 Genetic Pathology Evaluation Centre, University of British Columbia, Vancouver V6H 3Z6, BC, Canada; 2 Department of Pathology, Herlev and Gentofte University Hospital, 2730 Herlev, Denmark; 3 Danish Breast Cancer Cooperative Group, Rigshospitalet, Copenhagen University Hospital, 2100 Copenhagen, Denmark; 4 Centre for Regenerative Medicine and Stem Cell Research, Aga Khan University, Karachi 74800, Pakistan; 5 Department of Surgical Pathology, Zealand University Hospital, 4000 Roskilde, Denmark

**Keywords:** lymphocyte biomarkers, tumor immune microenvironment, immuno-oncology, breast cancer, adjuvant chemotherapy, cyclophosphamide, immunohistochemistry

## Abstract

**Simple Summary:**

Apart from the direct killing of cancer cells, cyclophosphamide-based chemotherapy has been shown to induce an antitumor immune response, and is being used in combination with immunotherapies in cancer care. We assessed the interaction of chemotherapy with immune biomarkers expressed on primary tumor tissue from a randomized phase III clinical trial, and confirmed that the presence of tumor-infiltrating lymphocytes is linked to improved survival in premenopausal women with high-risk breast cancer, regardless of their treatment allocation. However, immune biomarkers including tumor-infiltrating lymphocytes do not predict extra benefit from cyclophosphamide chemotherapy. This finding applies across the major molecular subgroups, including non-luminal and basal breast cancers that tend to be more immunogenic, and are often considered the most suitable subsets for receiving immunotherapy.

**Abstract:**

Recent studies have shown that immune infiltrates in the tumor microenvironment play a role in response to therapy, with some suggesting that patients with immunogenic tumors may receive increased benefit from chemotherapies. We evaluated this hypothesis in early breast cancer by testing the interaction between immune biomarkers and chemotherapy using materials from DBCG77B, a phase III clinical trial where high-risk premenopausal women were randomized to receive chemotherapy or no chemotherapy. Tissue microarrays were evaluated for tumor-infiltrating lymphocytes (TILs) assessed morphologically on hematoxylin and eosin-stained slides, and by immunohistochemistry for CD8, FOXP3, LAG-3, PD-1 and PD-L1. Following REMARK reporting guidelines, data analyses were performed according to a prespecified statistical plan, using 10-year invasive disease-free survival as the endpoint. Differences in survival probabilities between biomarker groups were evaluated by Kaplan–Meier and Cox proportional hazard ratio analyses and prediction for treatment benefit by an interaction test. Our results showed that stromal TILs were associated with an improved prognosis (HR = 0.93; *p*-value = 0.03), consistent with previous studies. However, none of the immune biomarkers predicted benefit from chemotherapy in the full study set nor within major breast cancer subtypes. Our study indicates that primary tumors with higher immune infiltration do not derive extra benefit from cyclophosphamide-based cytotoxic chemotherapy.

## 1. Introduction

Breast cancer is the most common malignancy in women worldwide [1]. Adjuvant cytotoxic chemotherapy remains the cornerstone of systemic treatment for high-risk early-stage breast cancers and has been instrumental in improving outcomes for these women [2]. Clinicopathological risk factors including age, disease stage, hormone receptor and human epidermal growth factor receptor-2 (HER2) status largely define the chemotherapy indication [2]. Recent findings have shown that chemotherapy can boost antitumor immunity through promoting immunogenic cell death and by blocking the immune evasion strategies [3]. In particular, as shown in preclinical [4,5,6,7] and in clinical studies [8,9,10], cyclophosphamides have been implicated in enhancing antitumor immune responses through releasing proinflammatory cytokines, recruiting dendritic cells, cytotoxic T cells, and natural killer cells into the tumor environment while concurrently decreasing levels of immune-suppressive regulatory T cells.

With the advent of immunotherapy, the immune-modulating features of conventional cytotoxic chemotherapies have received considerable attention in recent years. Breast cancer is not a particularly immunogenic malignancy and therefore combining immune checkpoint inhibitors with immune-augmenting modalities such as chemotherapy is a strategy under active investigation; for example, pembrolizumab has been granted approval for use with chemotherapy in high-risk, early-stage triple-negative breast cancer [11]. Other clinical trials investigating the effectiveness of combining chemotherapy with immunotherapy have shown improvements in pathological complete responses in advanced and in early-stage breast cancers [12,13,14].

Several studies highlight the important role of the immune microenvironment in response to therapies [15]. Tumor-infiltrating lymphocytes (TILs), key components of the adaptive immune system, have both prognostic and predictive value in breast cancer [16,17,18,19,20,21], though the extent of received benefits varies depending on molecular subtype. Estrogen receptor (ER)-negative breast cancers, including triple-negative and HER2-positive subtypes, have higher levels of immune infiltration when compared to ER-positive (luminal) breast cancers [22,23]. Although higher levels of TILs are generally associated with better outcomes, different subsets of lymphocytes (as defined by immune biomarkers) can have different impacts on survival [24]. Regulatory T cells characterized by FOXP3 (forkhead box protein 3) expression have immunomodulatory effects that can support cancer progression [25]. On the other hand, CD8+ cytotoxic T cells are associated with favorable prognosis in ER-negative breast cancers [26,27,28]. Moreover, CD8+TILs have been suggested to be predictive of anthracycline benefit in ER-negative breast cancers [27], whereas low sTILs predict superiority of trastuzumab over lapatinib in a formal prospective–retrospective analysis of the MA.31 trial of metastatic HER2-positive patients [29]. Additionally, immune checkpoint biomarkers such as LAG-3 (lymphocyte activation gene-3), PD-1 (programmed cell death protein 1), and PD-L1 (programmed death ligand 1) may also have prognostic value in breast cancer [30,31], and PD-L1 expression on immune cells is being used as a predictive biomarker for immune checkpoint inhibitors in triple-negative breast cancers [32].

Taken together, these studies suggest that pre-existing immune infiltrates in the primary tumor microenvironment might predict additional benefit from immune modulating chemotherapies such as cyclophosphamide. To test this hypothesis, we used archived excisional tumor materials from the Danish Breast Cancer Cooperative Group (DBCG)77B phase III clinical trial. This randomized trial has both chemotherapy and no chemotherapy arms in high-risk early breast cancer patients, thus providing an ideal setting to test our hypothesis [33]. The primary objective was to evaluate the predictive capacity of lymphocytic subsets, including stromal TIL levels and CD8+ lymphocytes as principal biomarkers in the full study set. The prespecified secondary objectives were designed to investigate (a) the primary predictive hypothesis in the higher risk non-luminal A subtypes, (b) prognostic relevance of the primary biomarkers in the full study set and non-luminal A cohort, and (c) the capacity of secondary biomarkers (FOXP3, LAG-3, and PD-1 expressing lymphocytes, and PD-L1+ immune cells) to predict response to cyclophosphamide-based chemotherapies in the full study set and non-luminal A cohort.

## 2. Materials and Methods

### 2.1. Study Cohort

**Figure 1:**
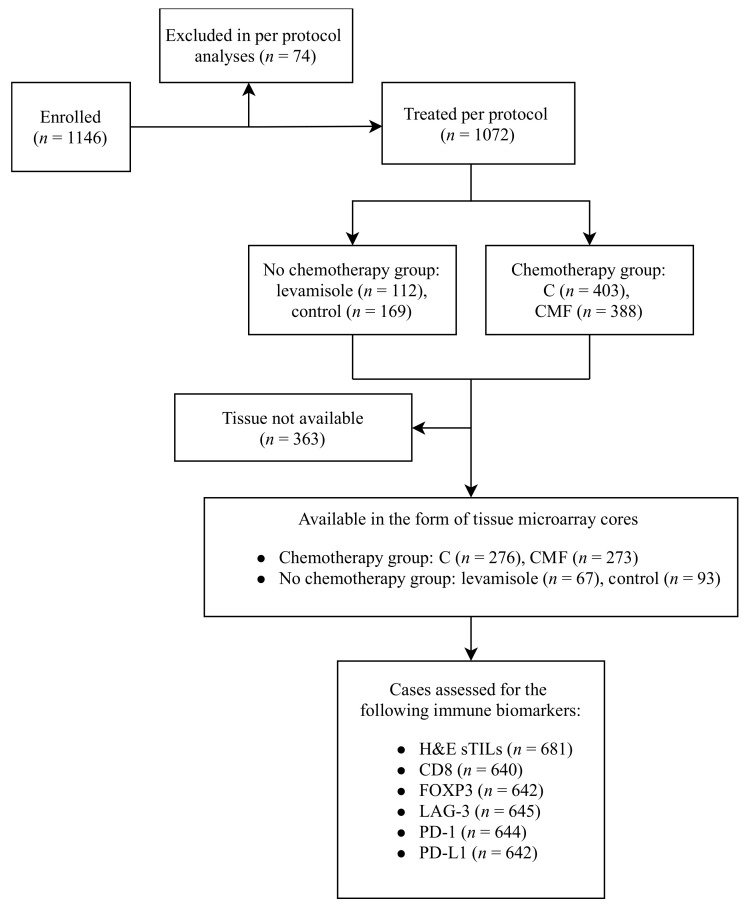
CONSORT diagram of DBCG77B phase III clinical trial immune biomarker study.

The DBCG77B clinical trial included premenopausal women diagnosed with high risk, non-metastatic invasive breast cancer (tumor size >5 cm, axillary nodal involvement or invasion of deep fascia) during 1977–1983. Patients were randomized into four treatment arms receiving either intravenous cyclophosphamide, methotrexate, and 5-fluorouracil (CMF), the single agent oral cyclophosphamide (C), levamisole (a non-chemotherapy antiparasitic drug), or no systemic therapy. All patients underwent mastectomy with axillary dissection (level I or II) and none of the patients received endocrine therapy as per the trial protocol. However, radiation to the chest wall and axilla was administered concomitantly with chemotherapy in the adjuvant setting to all enrolled patients [33]. The levamisole and the control arms were discontinued following the results of the first interim analyses showing high recurrence rates for both, and significant bone marrow suppression-related side effects amongst levamisole-treated patients [34]. Of 1146 patients enrolled in the trial, 1072 were included in the per protocol analysis. Patients who did not receive or discontinued the recommended trial intervention were excluded. The trial results were reported 25 years after the completion of recruitment with a 10-year median follow up period. Patients receiving chemotherapy regimens (C and CMF) had significantly superior survival when compared to the no chemotherapy arm. For the purpose of the present study, the four treatment arms were collapsed into ‘chemotherapy versus no chemotherapy groups’ based on the following rationale: given that the 10-year invasive disease-free survival (IDFS) rate for 403 patients who received C and 388 patients who received CMF was similar (55.5 and 48.8% respectively), they were combined into the ‘chemotherapy group’. Likewise, as the 10-year IDFS rates for 112 patients who had received levamisole (35.2%) and 169 patients in the no treatment group (38.6%) were similar, and levamisole is now considered inactive in cancer therapy, these groups were merged into the ‘no chemotherapy group’ [33]. The study was conducted in accordance with the Reporting Recommendations for Tumor Marker Prognostic Studies (REMARK) guidelines [35]. The CONSORT diagram illustrating the study design is shown in Figure 1.

### 2.2. Immunohistochemical Staining and Assessment

This study received ethics approval from the Biomedical Research Ethics Committee of the Capital Region of Denmark and the University of British Columbia BC Cancer Research Ethics Board (H-15012740, H17-01385). Biomarkers were immunostained on existing tissue microarrays, previously constructed from surgically excised primary tumors. Each case was represented by 2–3 cores, measuring 2 mm in diameter, with the full trial cohort spread over 24 tissue microarray blocks. Tissue microarray slides were created by sectioning at 4 µm and staining with hematoxylin and eosin (H&E) or by immunohistochemistry (IHC) for CD8 (1:50, mouse monoclonal, clone C8/144B, Agilent Technologies, Santa Clara, CA, USA), FOXP3 (1:20, mouse monoclonal, clone 236A/E7, Abcam, Cambridge, UK), LAG-3 (1:100, mouse monoclonal, clone 17B4, Abcam, Cambridge, UK), PD-1 (mouse monoclonal, clone NAT105, Cell Marque, Rocklin, CA, USA), and PD-L1 (1:100, rabbit monoclonal, clone SP142, Roche Diagnostics, Laval, QC, Canada) as previously described [36].

All biomarkers except for PD-L1 were stained at the Genetic Pathology Evaluation Centre (Vancouver, BC, Canada) on the Ventana Discovery Ultra autostainer. PD-L1 was stained at the Deeley Research Centre (Victoria, BC, Canada) [36]. Stained slides were scanned using a Leica Aperio AT2 scanner (Leica Biosystems, Concord, Canada). Representative photomicrographs are shown in Supplementary Figure S1.

CD8, FOXP3, LAG-3, and PD-1 were scored by DG, and PD-L1 and stromal TILs on H&E-stained sections were assessed by ESS as trained pathologists blinded to the clinical data. The absolute number of positive cells per tissue core was reported for CD8, FOXP3, LAG-3, and PD-1 as intraepithelial TILs (iTIL) when they are located inside of carcinoma nests in close contact with the malignant cells, and stromal TILs (sTIL) as those present in the stromal tissue between epithelial nests. PD-L1 was reported as per the manufacturer’s protocol, as the percentage of the tumor area occupied by the immune cells expressing PD-L1 [37]. Morphologically assessed sTILs on H&E-stained slides were reported as the percentage of the stromal compartment infiltrated by mononuclear immune cells, following the recommendations of the International TILs Working Group [38].

For each case with duplicate or triplicate cores, an average score was calculated. For IHC-assessed biomarkers, predefined cutoff points were locked down prior to data analyses based on those used in previous studies [26,31,37,39]. H&E sTIL was evaluated as a continuous variable for the primary objective as well as a categorical variable in prespecified exploratory analyses, using a pre-validated cutoff point close to the median value [29]. 

The tissue microarrays from this trial have been previously stained and scored for other biomarkers including ER (Clone SP1, Thermo Scientific, Waltham, MA, USA), PR (Clone 1E2, Ventana Medical Systems, Tucson, AZ, USA), HER2 (Clone SP3, Abcam, Cambridge, UK), CK5 (Clone XM26, Abcam, Cambridge, UK), EGFR (Clone EP22, Epitomics, Burlingame, USA), and Ki67 (Clone MIB-1, Agilent Technologies, Santa Clara, USA) for defining IHC-based breast cancer intrinsic subtypes, as previously published [40]. Briefly, IHC subtypes were defined as luminal A (ER and/or PR > 1%, HER2-negative with PR > 20% and Ki67 < 14%), luminal B (ER and/or PR > 1%, and (PR ≤ 20% or HER2+ or Ki67 ≥ 14%)), HER2-positive (ER− and PR− and HER2+), and basal (ER/PR/HER2 triple-negative and (CK5+ or EGFR+)). For the purpose of this study, we focused on the IHC-defined non-luminal A subset that combines the luminal B, HER2-positive and basal subtypes. Additional prespecified exploratory analyses were also performed in non-luminal (i.e., HER2-positive + basal) and basal subsets. 

### 2.3. Statistical Analysis

Statistical analyses were independently performed by the DBCG Data Center using a hypothesis driven, prespecified statistical plan. The Chi-squared test was used to analyze the associations between biomarkers and clinicopathological features as well as differences between included and excluded patients (excluding unknowns). IDFS was defined as the end point indicating the probability of survival without invasive breast cancer-related events at contralateral, locoregional or distant sites, non-breast primary malignancies or death irrespective of the cause, and was estimated using the Kaplan–Meier method. Univariate and multivariate analyses were performed using Cox proportional hazards regression models to calculate the unadjusted and adjusted hazard ratios (HR) and their corresponding confidence intervals (CI). The following factors were considered in the multivariate modeling: age (<40 years vs. ≥40 years), tumor size (0–2 cm, >2–5 cm, and >5 cm), the number of involved axillary lymph nodes (0–3, 4–9, and >9 positive lymph nodes; these categories were collapsed into ≤9 or >9 positive lymph nodes), histological type and grade (ductal Grade 1 and unknowns, 2, 3, and other histologic types), treatment regimen (chemotherapy (C + CMF arms) vs. no chemotherapy (control + levamisole arms)) and biomarkers in separate models. Invasion of the deep fascia was of no significance and was not included. No multivariate analysis was performed for the basal subset due to the limited number of events, and for the non-luminal subset the model was reduced. H&E sTIL were included as a continuous as well as a categorical variable, and other biomarkers were categorical. Functional form and proportional hazards assumptions were assessed by residuals and by including a time-dependent component in the model for each covariate. The hazard ratios for histological type and tumor grade were not proportional; therefore, stratification was used. For PD-L1, separate estimates were included according to the time since randomization. The Wald test for interaction was used to assess heterogeneity. All *p*-values are 2-tailed and are reported without adjusting for the multiple comparisons and a value of less than 0.05 was considered statistically significant. DBCG Statistical Office performed the statistical analyses using the SAS Enterprise Guide Version 7.15 software program package (SAS Institute, Cary, NC, USA). 

## 3. Results

### 3.1. Biomarker Distribution and Clinicopathological Factors

**Figure 2:**
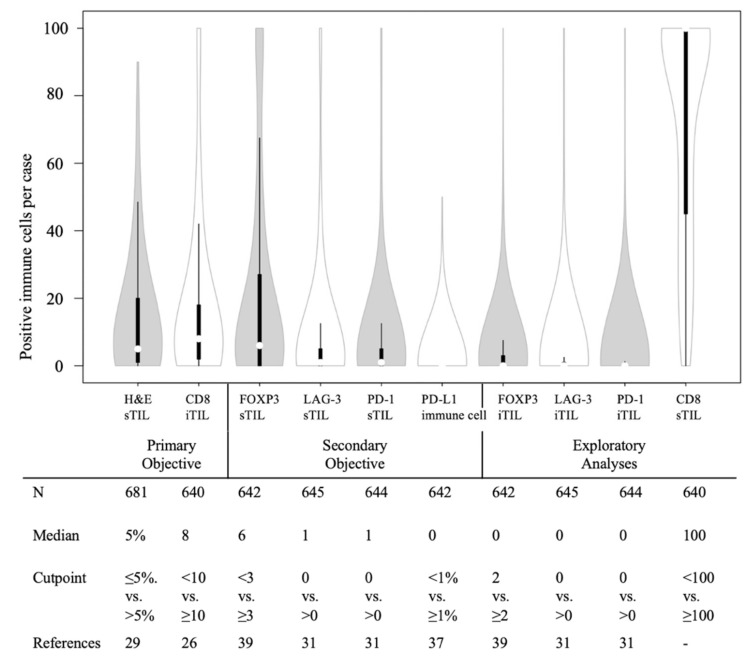
Violin plot showing the distribution of immune biomarkers, with the table summarizing the median values and predefined cut points, obtained from previous studies. White dots represent the median value for each marker. H&E sTIL was included in the primary objective as a continuous variable and in the exploratory analysis as a categorical variable. Cutoff point for CD8 iTIL was adjusted for the core size differences between this study (2.0 mm) and previous papers (0.6 mm). Median value was used as a cutoff point for CD8 sTILs. N: number of evaluable cases, H&E: hematoxylin and eosin, sTIL: stromal tumor-infiltrating lymphocyte, iTIL: intraepithelial tumor-infiltrating lymphocyte.

709 of the patients included in per protocol analysis also had tissue available for this study. Cores with insufficient tissue or less than 50 invasive carcinoma cells were considered unevaluable for biomarker assessment and therefore excluded from the analyses. Differences in the baseline clinicopathological characteristics between the patients included (*n* = 681) in the current study set or excluded according to the original trial per protocol analysis can be seen in Supplementary Table S1. Distribution of biomarkers including the numbers of evaluable cases per biomarker, median values and pre-validated cutoff points are shown in Figure 2. 

In both the chemotherapy and no chemotherapy groups, more than half of the patients had lower levels of H&E sTIL, iTILs expressing CD8, FOXP3, LAG-3, and PD-1, and PD-L1+ immune cells (most of which are macrophages) as defined by predetermined cutoff points (Supplementary Table S2). 

A significant association was detected between the level of immune infiltrates and age, tumor size, histological type, and tumor grade (Table 1). PD-1+ sTILs were more common among younger patients (*p*-value = 0.009). Lower levels of CD8+ iTILs and LAG-3+ sTILs were more prevalent in smaller tumors (<20 mm). However, higher levels of FOXP3+ regulatory sTILs were seen in tumors smaller than 50 mm (*p*-value < 0.05). Grade III tumors showed higher frequencies of TILs (H&E sTILs; CD8+ iTILs; FOXP3, LAG-3, and PD-1+ sTILs) but lower frequencies of PD-L1 negative immune cells when compared to grade I and II. By histological type, medullary breast cancers not surprisingly exhibited higher levels of CD8 iTILs and LAG-3 sTILs, whereas lobular carcinomas had lower levels of these immune cells compared to the other subtypes (*p*-value = 0.03). ER-negative tumors displayed significantly higher levels of H&E sTIL, CD8 iTIL, LAG-3 sTIL, PD-1 sTIL and PD-L1 immune cells (*p*-value < 0.05). Moreover, lower levels of H&E sTIL, and LAG-3 sTIL, and PD-1 immune cells were observed in patients with HER2-negative tumors (*p*-value < 0.05). Other clinicopathological factors and their associations with immune biomarkers are highlighted in Supplementary Table S3. The number of events according to immune biomarkers and treatment regimens are provided in Supplementary Table S4. 

### 3.2. Prognostic Value of the Primary Immune Biomarkers in the Full Study Set 

**Figure 3:**
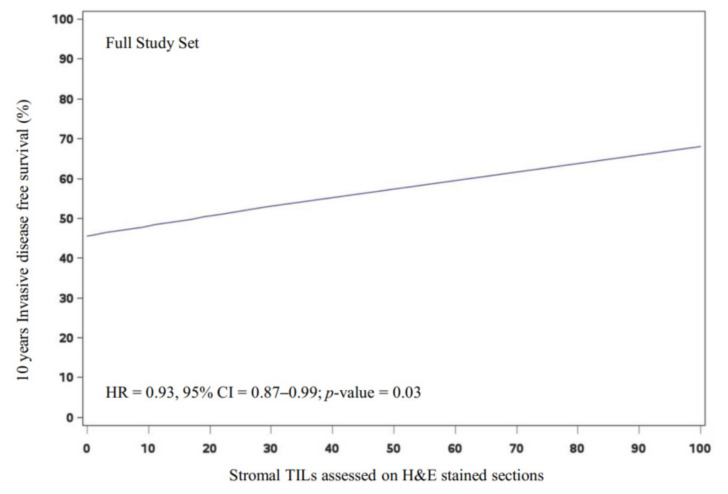
Prognostic significance of H&E sTIL in the full study set. Invasive disease-free survival plot and unadjusted hazard ratio for a 10-point difference in continuous H&E sTIL score (expressed as percentage) for the full study set (*n* = 681), using 10-year IDFS as the end point. HR: hazard ratio, CI: confidence interval, H&E: hematoxylin and eosin, IDFS: invasive disease-free survival, sTIL: stromal tumor-infiltrating lymphocyte.

In the full study set, the presence of increasing levels of tumor-infiltrating lymphocytes (sTILs as assessed on H&E sections) is associated with a significant improvement in breast cancer survival (10 years IDFS), with a 10-point difference in the continuous values in univariate (HR = 0.93, 95% CI = 0.87–0.99; *p*-value = 0.03) and multivariate analyses (HR = 0.93, 95% CI = 0.86–0.99; *p*-value = 0.03) (Figure 3). However, in the non-luminal A subgroup, differences did not reach statistical significance (multivariate HR = 0.94, 95% CI = 0.88–1.02; *p*-value = 0.12). Furthermore, the presence of high levels of CD8+ iTILs was not associated with a significant survival advantage in the full study set nor in the non-luminal A subgroup. Likewise, H&E sTILs when evaluated as a categorical variable in an exploratory analysis were not prognostic (Supplementary Figure S2). 

### 3.3. Predictive Value of Immune Biomarkers for Chemotherapy Benefit 

**Figure 4:**
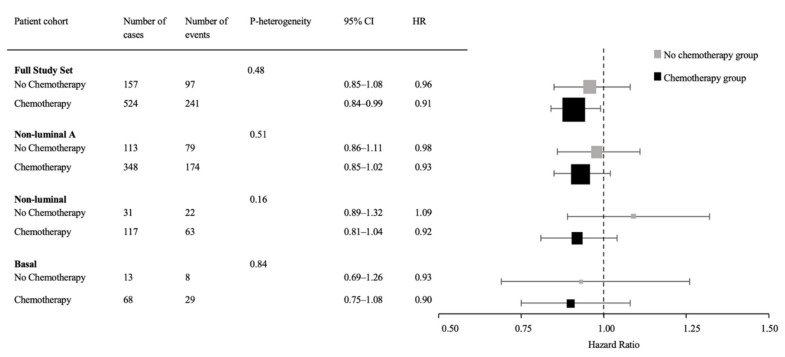
Forest plot showing adjusted hazard ratios and corresponding 95% confidence intervals for a 10-point difference in continuous H&E sTIL according to the treatment regimens. These analyses were performed for the full study set (primary objective), in the non-luminal A subset (secondary objective), and in the basal and non-luminal subsets (exploratory). Due to the limited number of the events, unadjusted estimates are presented for the basal subset. The box sizes represent the sample size (number of the cases in each subgroup analysis). CI: confidence interval, HR: hazard ratio, H&E: hematoxylin and eosin, sTIL: stromal tumor-infiltrating lymphocyte.

When compared to the no chemotherapy group, the chemotherapy arm was associated with significantly improved IDFS in the full study set (*n* = 681) in the multivariate analysis (HR = 0.55, 95% CI = 0.43–0.70; *p*-value < 0.0001). The primary prespecified hypothesis in the statistical plan was to assess the capacity of H&E sTIL (as a continuous variable) and CD8+ iTILs (as a categorical variable) to predict the benefit of chemotherapy (with the same analysis in the non-luminal A subset defined as a secondary objective). The multivariate analyses did not reveal a significant predictive value for H&E-assessed sTILs in any breast cancer subgroup (Figure 4). Similarly, CD8+iTILs did not demonstrate significant values in predicting benefit from chemotherapy in the full study set nor in the non-luminal A subset (Table 2). Although the presence of high CD8+iTILs was predictive of chemotherapy benefit (HR = 0.33, 95% CI = 0.16–0.65; *p*-value for heterogeneity = 0.03) in the non-luminal group, these results should be interpreted with caution due to the small number of the events in the no-chemotherapy group with low CD8+iTILs. The predictive exploratory analysis in the basal subset was not performed due to the limited number of events. 

For the additional secondary immune biomarkers, analyses showed a significant improvement in IDFS only in tumors with high PD-1 sTIL infiltration (HR = 0.42, 95% CI = 0.30–0.59 versus HR = 0.71, 95% CI = 0.49–1.03; *p*-value for heterogeneity = 0.04) when analyzed for the full study set. However, these significant results were not maintained when the analysis was restricted to the non-luminal A subgroup. None of the other secondary immune biomarkers expressed on intraepithelial or stromal lymphocytes were predictive of an enhanced benefit from chemotherapy in the full study set nor in the non-luminal A subgroup (Table 2). Overall, our analyses find that the immune biomarkers did not predict enhanced cyclophosphamide-based chemotherapy benefit in the DBCG77B randomized trial.

## 4. Discussion

Here we have assessed the clinical relevance of tumor immune infiltrates in predicting response to cyclophosphamide-based chemotherapy using materials from a phase III randomized clinical trial for which 10 year clinical outcome data were available. The findings from this formal prospective–retrospective translational study, within the limits of its power, do not support the hypothesis that pre-existing immune infiltrates predict enhanced benefit from cyclophosphamide-based chemotherapy. This finding is particularly relevant in the context of triple-negative/basal subsets and non-luminal subgroups in which tumors often demonstrate (variably dense) immune infiltrates [41]. The results of our study are consistent with a previous finding from a French group combining two breast cancer trials randomizing to anthracycline-based vs. no chemotherapy, which found that even though TIL counts on primary tumor specimens are prognostic (particularly in triple-negative and HER2-positive breast cancer), they do not predict benefit from anthracycline chemotherapy [42]. Our findings extend this observation to non-anthracycline (cyclophosphamide) chemotherapy, and to multiple immune IHC biomarkers beyond simple H&E TIL counts.

Although a significant predictive interaction was seen for the CD8 iTIL in the non-luminal subset (which includes basal and HER2-positive subtypes), this exploratory finding needs to be interpreted with caution due to the limited number of cases and events occurring for this analysis, its borderline *p*-value in the context of an exploratory finding, and the different behavior of the HER2-positive patients with CMF chemotherapy in DGCG77B [43]. 

A possible explanation for the negative result of the primary tested hypothesis may be that antitumor immunity induced by cyclophosphamide-based chemotherapy is dependent on dose and chemotherapy schedules [44]. Ghiringhelli et al. reported that the inhibition of regulatory T cells by cyclophosphamide is only achieved when it is administered as a metronomic treatment in low doses (20–100 mg per day). Higher doses of cyclophosphamide can deplete all the lymphocyte subpopulations [9,45]. Even though cyclophosphamide dosage in the chemotherapy arm of the DBCG77B clinical trial fell within the appropriate ‘low’ dose range (130 mg/m2 in oral C and 80 mg/m2 in CMF), it was not administered in a metronomic schedule (cyclophosphamide was given on days 1 to 14 every 4 weeks for 12 cycles). Though our study was based on pre-treatment surgical excisions, the apparent lack of interaction between immune infiltration and chemotherapy benefit could potentially be related to these differences in treatment schedule and dosages. 

There are ongoing investigations on different combinations of immunotherapy and chemotherapy, particularly in the neoadjuvant setting in triple-negative breast cancers [46]. Encouraging results obtained from these trials support the value of chemotherapy in augmenting the host immune response. However, the antitumor immunity induced by neoadjuvant chemotherapy is carried out by new T cell clones rather than pre-existing ones, supporting our findings [47]. Moreover, different chemotherapeutic agents exert different mechanisms of action that can have distinct impacts on the tumor microenvironment. For instance, in the TONIC trial randomizing patients to receive nivolumab with different immunomodulatory chemotherapies, cohorts allocated to cisplatin and doxorubicin comprised the majority of responses compared to cyclophosphamide [48].

### Strengths and Limitations

Strengths of our study include using material from a randomized phase III clinical trial and long-term clinical follow up data. Most importantly, this older trial included a no-chemotherapy arm in relatively high-risk patients, something no longer ethically possible to assess in newer trials, that allowed for the assessment of the predictive value of immune biomarkers for chemotherapy with cyclophosphamide, a major backbone drug in most breast cancer regimens to this day. Another factor that provided strength to this study was compiling a pre-specified statistical plan with predetermined cutoff points and independently executed statistical analyses, following the REMARK guidelines [35]. 

It is plausible that study limitations may have affected our conclusions. Firstly, we used tissue microarrays for evaluating biomarkers. Although tissue microarrays will not completely capture the heterogeneity of the tumor microenvironment, they do facilitate assessing a large number of cases that would otherwise require enormous resources [49]. For this study, we used 2.0 mm tissue microarray cores (with two to three cores per case) that are in fact 11 times bigger than the regularly used 0.6 mm tissue microarray cores. Moreover, this study was underpowered to fully assess the predictive and prognostic values of immune biomarkers in breast cancer molecular subsets (specifically in the non-luminal and basal cohorts) partly due to the early closure of the no chemotherapy arm. Additionally, another drawback to this study is using pre-treatment excisions to infer the immune status of subclinical micrometastases. The micrometastases that are being targeted by adjuvant chemotherapy are subclones of the primary tumors and could exhibit a different tumor immune microenvironment.

Another limitation that may have contributed to the negative result in this paper is our approach to capture the immune biomarkers. Conventional yet widely used techniques such as staining the slides with the single marker IHC or H&E provides basic information about a complex tumor microenvironment. Although these approaches are affordable and accessible, utilizing novel high plex techniques to study the spatial relationships between malignant and immune compartments can potentially uncover new findings that we were unable to assess with these conventional methods [50]. Different studies would be required, for example, to evaluate the contribution of innate immune cells to antitumor responses induced by cytotoxic chemotherapies. Mouse model studies have shown that the combination of cyclophosphamide with immunotherapy potentiates innate immune responses through impacting macrophage, dendritic cell, and natural killer cell populations [51,52].

## 5. Conclusions

Immune infiltrates in the tumor microenvironment assessed by H&E are prognostic of improved IDFS in the full study set. However, no significant interaction was observed between any of the assessed immune biomarkers and chemotherapy benefit, including among major breast cancer subgroups, indicative of their lack of predictive capacity for cyclophosphamide-based adjuvant chemotherapy. 

## Figures and Tables

**Table 1:** Correlation of primary and secondary biomarkers with clinicopathological characteristics, histological subtypes and treatment groups. Percentage of cases within the given factor is shown in parentheses. Cases with unknown characteristics are not shown in the table. sTIL: stromal tumor-infiltrating lymphocyte, iTIL: intraepithelial tumor-infiltrating lymphocyte, H&E: hematoxylin and eosin.

	H&E sTIL		CD8 iTIL		FOXP3 sTIL		LAG-3 sTIL		PD-1 sTIL		PD-L1 Immune Cell	
	Low ≤5% *n* = 361 (%)	High >5% *n* = 320 (%)	Low <10 *n* = 355 (%)	High ≥10 *n* = 285 (%)	Low <3 *n* = 253 (%)	High ≥3 *n* = 389 (%)	Negative 0 *n* = 314 (%)	Positive >0 *n* = 331 (%)	Negative 0 *n* = 307 (%)	Positive >0 *n* = 337 (%)	Negative <1% *n* = 512 (%)	Positive ≥1% *n* = 130 (%)
Age (years)												
≤50	266 (52)	245 (48)	273 (56)	211 (44)	185 (38)	299 (62)	246 (51)	241 (49)	217 (45)	268 (55)	389 (81)	94 (19%)
>50	95 (56)	75 (44)	82 (53)	74 (47)	68 (43)	90 (57)	68 (43)	90 (57)	90 (57)	69 (43)	123 (77)	36 (23%)
*p*-value	0.386		0.401		0.282		0.102		0.009		0.387	
Tumor size (mm)												
0–20	110 (52)	101 (48)	123 (65)	67 (35)	81 (42)	111 (58)	110 (58)	80 (42)	92 (48)	101 (52)	158 (82)	34 (18)
21–50	184 (53)	160 (47)	168 (52)	158 (48)	110 (34)	216 (66)	149 (45)	182 (55)	159 (48)	170 (52)	253 (78)	73 (22)
>50	67 (55)	54 (45)	63 (53)	56 (47)	61 (51)	58 (49)	54 (45)	65 (55)	55 (47)	62 (53)	98 (82)	21 (18)
*p*-value	0.850		0.011		0.002		0.013		0.968		0.333	
Histological type												
Ductal	310 (52)	281 (48)	306 (55)	252 (45)	214 (38)	345 (62)	267 (47)	297 (53)	264 (47)	298 (53)	441 (79)	119 (21)
Lobular	24 (60)	16 (40)	27 (73)	10 (27)	20 (54)	17 (46)	24 (67)	12 (33)	19 (53)	17 (47)	35 (92)	3 (8)
Medullary	4 (36)	7 (64)	2 (22)	7 (78)	6 (67)	3 (33)	2 (22)	7 (78)	2 (22)	7 (78)	5 (63)	3 (37)
Other	18 (58)	13 (42)	16 (57)	12 (43)	10 (34)	19 (66)	17 (61)	11 (39)	18 (62)	11 (38)	25 (89)	3 (11)
*p*-value	0.494		0.033		0.082		0.027		0.156		0.072	
Grade												
1	67 (57)	50 (43)	72 (67)	35 (33)	45 (42)	61 (58)	69 (65)	37 (35)	59 (56)	47 (44)	93 (87)	14 (13)
2	195 (55)	162 (45)	187 (55)	152 (45)	137 (40)	204 (60)	171 (50)	174 (50)	166 (49)	175 (51)	278 (81)	66 (19)
3	48 (41)	69 (59)	46 (41)	65 (59)	31 (28)	80 (72)	27 (24)	86 (76)	38 (33)	76 (67)	69 (64)	39 (36)
*p*-value	0.019		0.0006		0.042		<0.0001		0.002		<0.0001	
ER status												
<1%	62 (37)	107 (63)	67 (42)	92 (58)	54 (35)	102 (65)	41 (26)	119 (74)	61 (38)	98 (62)	93 (61)	60 (39)
≥1%	295 (59)	207 (41)	288 (59)	203 (41)	196 (39)	302 (61)	273 (55)	221 (45)	250 (50)	246 (50)	423 (86)	71 (14)
*p*-value	<0.0001		0.0003		0.288		<0.0001		0.008		<0.0001	
HER2 status												
0, 1+, 2+	307 (57)	231 (43)	291 (56)	226 (44)	213 (41)	310 (59)	275 (53)	245 (47)	252 (48)	271 (52)	435 (84)	80 (16)
3+	50 (36)	87 (64)	63 (47)	71 (53)	40 (30)	92 (70)	39 (29)	96 (71)	60 (45)	74 (55)	84 (63)	50 (37)
*p*-value	<0.0001		0.055		0.279		<0.0001		0.481		<0.0001	

**Table 2:** Adjusted hazard ratios (HR) and corresponding 95% confidence intervals (CIs) are shown for the chemotherapy versus no chemotherapy arms according to the biomarker status (low/negative and high/positive) in the full study set and in the non-luminal A subset. sTIL: stromal tumor-infiltrating lymphocytes, iTIL: intraepithelial tumor-infiltrating lymphocytes, H&E: hematoxylin and eosin.

Marker		HR	95% CI	P-Heterogeneity	HR	95% CI	P-Heterogeneity
		Full Study Set			Non Luminal A		
		Primary objective			Secondary objective		
CD8 iTILs	Low <10	0.50	0.36–0.70	0.54	0.46	0.31–0.68	0.68
	High ≥10	0.59	0.41–0.85		0.51	0.35–0.76	
Secondary objective							
FOXP3 sTIL	Low <3	0.49	0.33–0.72	0.48	0.39	0.25–0.62	0.22
	High ≥3	0.58	0.42–0.80		0.56	0.40–0.79	
LAG-3 sTIL	Negative 0	0.53	0.36–0.78	0.68	0.47	0.30–0.76	0.65
	Positive >0	0.59	0.43–0.81		0.54	0.39–0.76	
PD-1 sTIL	Negative 0	0.71	0.49–1.03	0.04	0.53	0.35–0.81	0.75
	Positive >0	0.42	0.30–0.59		0.49	0.34–0.70	
PD-L1	Negative <1%	0.58	0.43–0.77	0.30	0.52	0.38–0.72	0.48
	Positive ≥1%	0.43	0.26–0.71		0.42	0.25–0.71	
Exploratory analyses							
H&E (categorical)	Low ≤5%	0.54	0.40–0.75	0.95	0.49	0.34–0.71	0.65
	High >5%	0.55	0.38–0.79		0.55	0.37–0.82	
FOXP3 iTIL	Low <2	0.50	0.37–0.68	0.32	0.44	0.31–0.62	0.26
	High ≥2	0.64	0.42–0.97		0.60	0.39–0.94	
LAG-3 iTIL	Negative 0	0.62	0.45–0.84	0.27	0.56	0.39–0.79	0.41
	Positive >0	0.46	0.31–0.70		0.44	0.28–0.68	
PD-1 iTIL	Negative 0	0.62	0.46–0.83	0.11	0.55	0.39–0.77	0.37
	Positive >0	0.40	0.25–0.62		0.42	0.26–0.69	
CD8 sTIL	Low <100	0.47	0.33–0.67	0.36	0.42	0.28–0.63	0.40
	High ≥100	0.59	0.41–0.83		0.53	0.36–0.77	

## Data Availability

Biomarker data are available on request from the corresponding author. Due to institutional restrictions, the clinical data are not publicly available but can be provided to qualified researchers through Danish Breast Cancer Group: dbcg.rigshospitalet@regionh.dk.

## Supplementary Materials

The following supporting information can be downloaded at: https://www.mdpi.com/article/10.3390/cancers14153808/s1, Table S1: Comparison of the baseline clinicopathological characteristics of the included and excluded cases from the DBCG77B phase III clinical trial; Table S2. Distribution of immune biomarkers according to the treatment regimens; Table S3. Correlation between patient characteristics and primary and secondary immune biomarkers; Table S4. Number of the events according to immune biomarkers and treatment regimens in the full study set; Figure S1. Photomicrographs of tumor sections stained with the immune biomarkers in DBCG77B study; Figure S2. Kaplan Meier curves showing the prognostic significance of CD8iTIL and H&E sTIL in the full study set and non-luminal A cohort.
