# Supplementary material for: Neither Tumor-Infiltrating Lymphocytes nor Cytotoxic T Cells Predict Enhanced Benefit from Chemotherapy in the DBCG77B Phase III Clinical Trial
Source: Cancers (Basel). 2022 Aug 5;14(15):3808. doi: 10.3390/cancers14153808 (PMC9367267; doi:10.3390/cancers14153808)
Supplement: Supplementary file 1 [file cancers-14-03808-s001.zip › cancers-1829375-supplementary.pdf]

Supplementary Materials: Neither Tumor Infiltrating Lymphocytes nor Cytotoxic T Cells Predict Enhanced Benefit from Chemotherapy in the DBCG77B Phase III Clinical Trial

Elahe Shenasa, Elisabeth Specht Stovgaard, Maj-Britt Jensen, Karama Asleh, Nazia Riaz, Dongxia Gao, Samuel Leung, Bent Ejlertsen, Anne-Vibeke Laenkholm and Torsten O. Nielsen

Table S1. Comparison of the baseline clinicopathological characteristics of the included and excluded cases from the DBCG77B phase III clinical trial.

	Included cases *N*=681 (%)	Excluded cases *N*=465 (%)	Total	*p*-value
**Age**				0.44
≤40	165 (24)	102 (2)	267	
41-45	164 (24)	100 (21)	264	
46-50	182 (27)	134 (29)	316	
>50	170 (25)	129 (28)	299	
**Tumor size (micro), mm**				0.73
0-20	180 (26)	110 (24)	290	
21-50	277 (41)	151 (32)	428	
>50	92 (14)	56 (12)	148	
Unknown	132 (19)	148 (32)	280	
**Positive lymph nodes**				0.03
0	99 (14)	95 (20)	194	
1-3	394 (58)	250 (54)	644	
4+	188 (28)	120 (26)	308	
**Histological type**				0.47
Ductal	591 (87)	411 (88)	1002	
Lobular	40 (6)	18 (4)	58	
Medullary	11 (2)	6 (1)	17	
Other	31 (4)	23 (5)	54	
Unknown	8 (1)	7 (2)	15	
**Grade, ductal**				0.26
I	117 (20)	96 (23)	213	
II	357 (60)	225 (55)	582	
III	117 (20)	82 (20)	199	
Unknown	0 (0)	8 (2)	8	

Table S1. Included patients are those cases that are included in the original clinical trial per protocol analysis who have also H&E slides avaible for assessment. *p*-values derived after excluding unknowns. sTIL: stromal tumor infiltrating lymphocyte, H&E: hematoxylin and eosin.

Table S2. Distribution of immune biomarkers according to the treatment regimens .

A) All Patients.

Markers (predetermined cutpoints)	No chemotherapy group		Chemotherapy group	
	Low/ Negative (%)	High/ Positive (%)	Low/ Negative (%)	High/ Positive (%)
H&E sTIL (≤5%. vs. >5%)	86 (55)	71 (45)	275 (52)	249 (48)
CD8 iTIL (<10 vs. ≥10)	79 (54)	68 (46)	276 (56)	217 (44)
FOXP3 sTIL (<3 vs. ≥3)	59 (39)	91 (61)	194 (39)	298 (61)
LAG-3 sTIL (0 vs. >0)	68 (46)	81 (54)	246 (50)	250 (50)
PD-1 sTIL (0 vs. >0)	72 (48)	78 (52)	235 (48)	259 (52)
PD-L1 (<1% vs. ≥1%)	114 (75)	37 (25)	398 (81)	93 (19)
CD8 sTIL (<100 vs. ≥100)	62 (42)	85 (58)	250 (51)	243 (49)
FOXP3 iTIL (<2 vs. ≥2)	96 (64)	54 (36)	332 (67)	160 (33)

LAG-3 iTIL (0 vs. >0)	99 (66)	50 (34)	355 (72)	141 (28)
PD-1 iTIL (0 vs. >0)	108 (72)	42 (28)	365 (74)	129 (26)

B) Non luminal A (HER2 + basal + luminal B).

**Markers (predetermined cutpoints)**	**No chemotherapy group**		**Chemotherapy group**	
	**Low/ Negative (%)**	**High/ Positive (%)**	**Low/ Negative (%)**	**High/ Positive (%)**
H&E sTIL (≤5%. vs. >5%)	59 (52)	54 (48)	165 (47)	183 (53)
CD8 iTIL (<10 vs. ≥10)	53 (49)	56 (51)	168 (50)	169 (50)
FOXP3 sTIL (<3 vs. ≥3)	38 (34)	74 (66)	120 (36)	212 (64)
LAG-3 sTIL (0 vs. >0)	42 (38)	69 (62)	135 (40)	200 (60)
PD-1 sTIL (0 vs. >0)	47 (42)	65 (58)	140 (42)	195 (58)
PD-L1 (<1% vs. ≥1%)	77 (70)	33 (30)	253 (77)	77 (23)
CD8 sTIL (<100 vs. ≥100)	43 (39)	66 (61)	162 (48)	175 (52)
FOXP3 iTIL (<2 vs. ≥2)	69 (62)	43 (38)	203 (61)	129 (39)
LAG-3 iTIL (0 vs. >0)	66 (59)	45 (41)	215 (64)	120 (36)
PD-1 iTIL (0 vs. >0)	75 (67)	37 (33)	234 (70)	101 (30)

Table S2. Distribution of immune biomarkers according to the treatment regimens [chemotherapy (C+CMF) versus no chemotherapy] among A) all patients and B) Non luminal A patients (HER2 + basal + luminal B). sTIL: stromal tumor infiltrating lymphocyte, iTIL: intraepithelial tumor infiltrating lymphocyte, H&E: hematoxylin and eosin.

Table S3. Correlation between patient characteristics and primary and secondary immune biomarkers.

	**H&E sTIL**		**CD8 iTIL**		**FOXP3 sTIL**		**LAG-3 sTIL**		**PD-1 sTIL**		**PD-L1 immune cell**	
	Low ≤5% *N*=361 (%)	High >5% *N*=320 (%)	Low <10 *N*=355 (%)	High ≥10 *N*=285 (%)	Low <3 *N*=253 (%)	High ≥3 *N*=389 (%)	Negative 0 *N*=314 (%)	Positive >0 *N*=331 (%)	Negative 0 *N*=307 (%)	Positive >0 *N*=337 (%)	Negative <1% *N*=512 (%)	Positive ≥1% *N*=130 (%)
**Positive lymph nodes**												
0	56 (57)	43 (43)	53 (57)	40 (43)	37 (39)	57 (61)	49 (52)	45 (48)	49 (52)	45 (48)	77 (80)	19 (20)
1-3	201 (51)	193 (49)	208 (57)	158 (43)	141 (38)	229 (62)	192 (51)	181 (49)	169 (45)	203 (55)	292 (80)	73 (20)
4+	104 (55)	84 (45)	94 (52)	87 (48)	75 (42)	103 (58)	73 (41)	105 (59)	89 (50)	89 (50)	143 (79)	38 (21)
P-value	0.46		0.53		0.66		0.05		0.39		0.96	
**Molecular Subsets**												
Non-Luminal A	224 (49)	237 (51)	221 (50)	225 (50)	158 (36)	286 (64)	177 (40)	269 (60)	187 (42)	260 (58)	330 (75)	110 (25)
Non-Luminal	49 (33)	99 (67)	58 (41)	82 (59)	44 (32)	92 (68)	33 (24)	107 (76)	49 (35)	90 (65)	78 (59)	55 (41)
Basal	25 (31)	56 (69)	26 (34)	50 (66)	24 (32)	51 (68)	15 (20)	61 (80)	23 (30)	53 (70)	39 (56)	31 (44)
**Treatment Allocation**												
Chemotherapy	275 (52)	249 (48)	276 (56)	217 (44)	194 (39)	298 (61)	246 (50)	250 (50)	235 (48)	259 (52)	398 (81)	93 (19)
No Chemotherapy	86 (55)	71 (45)	79 (54)	68 (46)	59 (39)	91 (61)	68 (46)	81 (54)	72 (48)	78 (52)	114 (76)	37 (24)
P-value	0.61		0.63		1.00		0.40		0.93		0.14	

sTIL: stromal tumor infiltrating lymphocyte, iTIL: intraepithelial tumor infiltrating lymphocyte, H&E: hematoxylin and eosin.

Table S4. Number of the events* according to immune biomarkers and treatment regimens in the full study set.

A) All Patients.

**Marker**	**No chemotherapy group**		**Chemotherapy group**	
	**Low/ Negative (%)**	**High/ Positive (%)**	**Low/ Negative (%)**	**High/ Positive (%)**
H&E sTIL (≤5%. vs. >5%)	55 (57)	42 (43)	138 (57)	103 (43)
CD8 iTIL (<10 vs. ≥10)	49 (54)	42 (46)	131 (57)	99 (43)
FOXP3 sTIL (<3 vs. ≥3)	37 (40)	55 (60)	91 (40)	135 (60)

LAG-3 sTIL (0 vs. >0)	37 (41)	54 (59)	103 (45)	128 (55)
PD-1 sTIL (0 vs. >0)	40 (43)	52 (57)	111 (49)	115 (51)
PD-L1 (<1% vs. ≥1%)	68 (72)	26 (28)	188 (83)	39 (17)
CD8 sTIL (<100 vs. ≥100)	42 (46)	49 (54)	127 (55)	103 (45)
FOXP3 iTIL (<2 vs. ≥2)	59 (64)	33 (36)	149 (66)	77 (34)
LAG-3 iTIL (0 vs. >0)	55 (60)	36 (40)	165 (71)	66 (29)
PD-1 iTIL (0 vs. >0)	62 (67)	30 (33)	169 (75)	57 (25)

B) Non luminal A (HER2 + basal + luminal B).

Marker	No chemotherapy group		Chemotherapy group	
	Low/ Negative (%)	High/ Positive (%)	Low/ Negative (%)	High/ Positive (%)
H&E sTIL (≤5%. vs. >5%)	43 (54)	36 (46)	88 (51)	86 (49)
CD8 iTIL (<10 vs. ≥10)	39 (50)	39 (50)	90 (53)	79 (47)
FOXP3 sTIL (<3 vs. ≥3)	29 (37)	50 (63)	60 (36)	105 (64)
LAG-3 sTIL (0 vs. >0)	27 (35)	51 (65)	62 (37)	107 (63)
PD-1 sTIL (0 vs. >0)	33 (42)	46 (58)	68 (41)	99 (59)
PD-L1 (<1% vs. ≥1%)	54 (68)	25 (32)	131 (79)	35 (21)
CD8 sTIL (<100 vs. ≥100)	34 (44)	44 (56)	90 (53)	79 (47)
FOXP3 iTIL (<2 vs. ≥2)	50 (63)	29 (37)	102 (62)	63 (38)
LAG-3 iTIL (0 vs. >0)	44 (56)	34 (44)	114 (67)	55 (33)
PD-1 iTIL (0 vs. >0)	51 (65)	28 (35)	120 (72)	47 (28)

C) Basal.

Marker	No chemotherapy		Chemotherapy	
	Low/ Negative (%)	High/ Positive (%)	Low/ Negative(%)	High/ Positive (%)
H&E sTIL (≤5%. vs >5%)	2 (25)	6 (75)	11 (38)	18 (62)
CD8 iTIL (<10 vs. ≥10)	2 (25)	6 (75)	10 (38)	16 (62)

D) Non luminal (HER2 + basal).

Marker	No chemotherapy		Chemotherapy	
	Low/ Negative (%)	High/ Positive (%)	Low/ Negative(%)	High/ Positive (%)
H&E sTIL (≤5%. vs >5%)	9 (33)	13 (67)	23 (31)	40 (69)
CD8 iTIL (<10 vs. ≥10)	7 (41)	14 (59)	29 (37)	64 (63)

sTIL: stromal tumor infiltrating lymphocyte, iTIL: intraepithelial tumor infiltrating lymphocyte, H&E: hematoxylin and eosin.

* Events were defined according to the endpoint of this study: Invasive disease free survival was defined as the end point indicating the probability of survival without invasive breast cancer related events at contralateral, locoregional or distant sites, non-breast primary malignancies or death irrespective of the cause.

Figure S1. Photomicrographs of tumor sections stained with the immune biomarkers in DBCG77B study.

A) CD8

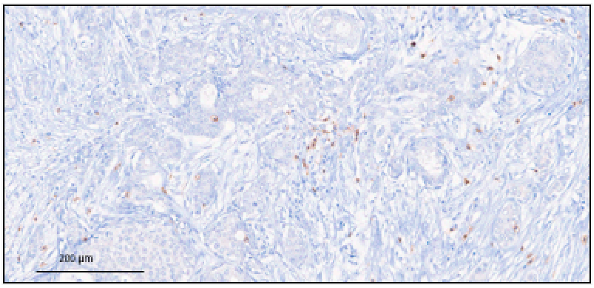

B) FOXP3

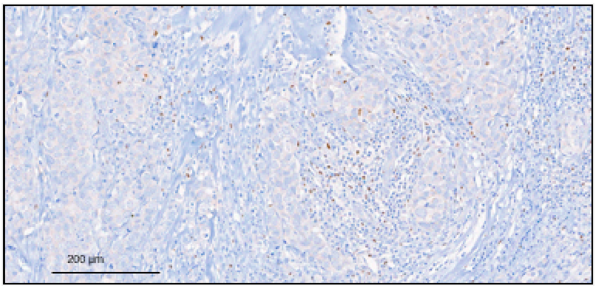

C) LAG-3

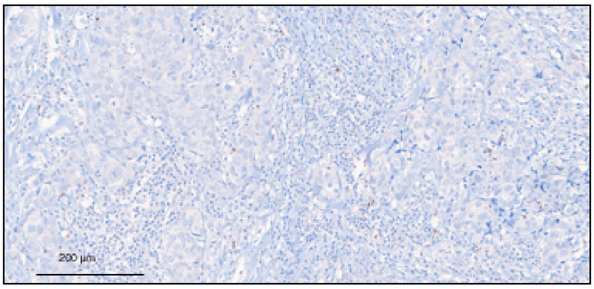

D) PD-1

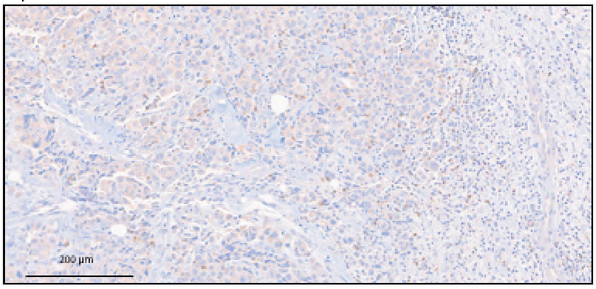

E) PD-L1

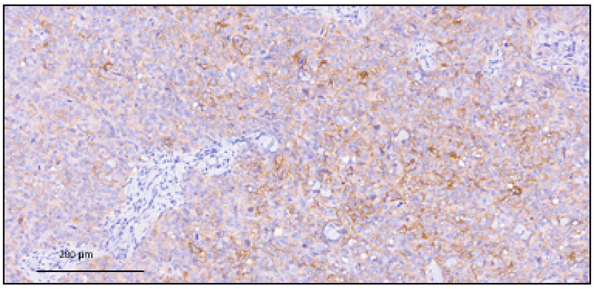

F) H&E

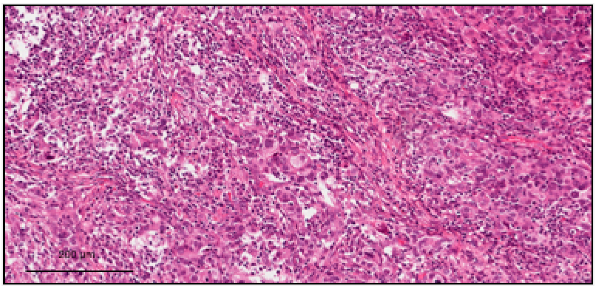

Figure S1. Immunohistochemical images of A) CD8 (1:50, clone C8/144B, Agilent Technologies, Santa Clara, USA), B) FOXP3 (1:20, clone 236A/E7, Abcam, Cambridge, UK), C) LAG-3 (1:100, clone 17B4, Abcam, Cambridge, UK), D) PD-1 (clone NAT105, Cell Marque, Rocklin, USA), E) PD-L1 (1:100, clone SP142, Roche Diagnostics, Laval, CA) and F) hematoxylin & eosin stained image for assessment of stromal tumor infiltrative lymphocytes (sTILs). All slides were stained on the Ventana Discovery Ultra autostainer at the Genetic Pathology Evaluation Centre (Vancouver, Canada) except for PD-L1 which was stained at the Victoria Deeley Research Centre (Victoria, Canada), as previously described[36]. Image examples are obtained from different tissue microarray cases, captured at the 20X magnification.

Figure S2. Kaplan Meier curves showing the prognostic significance of CD8iTIL and H&E sTIL in the full study set and non-luminal A cohort.

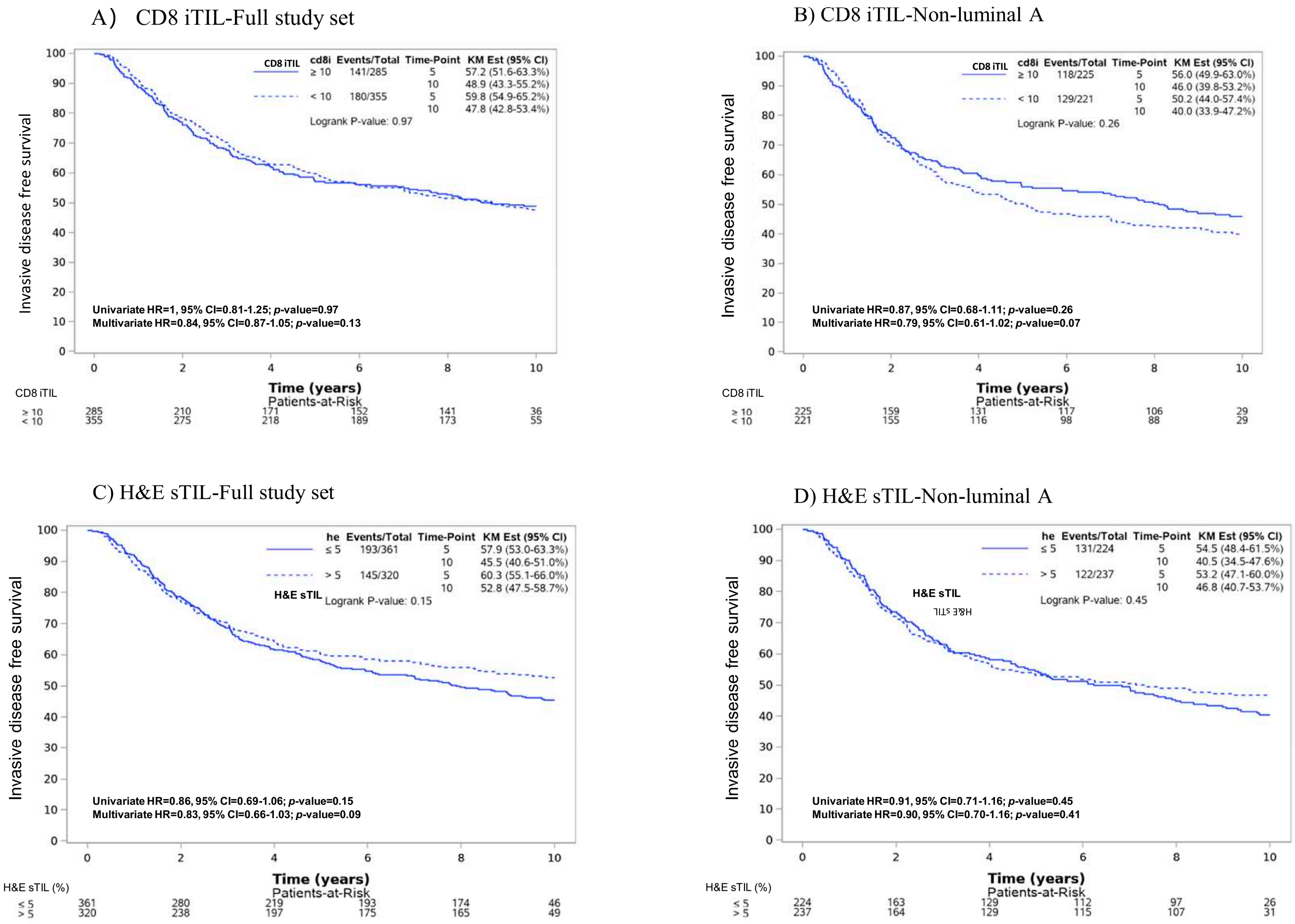

Figure S2. Kaplan Meier survival curves showing the probability of invasive disease free survival for CD8+ iTILs in the A) full study set and B) non luminal A subgroup; and H&E sTILs (categorical variable) in the C) full study set and D) non luminal A subset. HR: hazard ratio, CI: confidence interval, iTIL: intraepithelial tumor infiltrating lymphocyte, H&E: hematoxylin and eosin, sTIL: stromal tumor infiltrating lymphocyte.
